# Space Asymmetry Directs Preferential Sperm Entry in the Absence of Polarity in the Mouse Oocyte

**DOI:** 10.1371/journal.pbio.0040135

**Published:** 2006-04-25

**Authors:** Nami Motosugi, Jens-Erik Dietrich, Zbigniew Polanski, Davor Solter, Takashi Hiiragi

**Affiliations:** **1**Max-Planck Institute of Immunobiology, Freiburg, Germany; Osaka UniversityJapan

## Abstract

Knowledge about the mechanism that establishes embryonic polarity is fundamental in understanding mammalian development. In re-addressing several controversial claims, we recently proposed a model in which mouse embryonic polarity is not specified until the blastocyst stage. Before fertilization, the fully differentiated oocyte has been characterized as “polarized,” and we indeed observed that the sperm preferentially enters the polar body half. Here we show that preferential sperm entry is not due to an intrinsic polarity of the oocyte, since fertilization takes place uniformly when the zona pellucida is removed. We suggest that the term “asymmetry” denotes morphological differences, whereas “polarity” in addition implies developmental consequences. Thus, the mouse oocyte can be considered “asymmetric” but “non-polarized.” The penetration through the zona pellucida is also random, and a significant proportion of sperm binds to the oocyte membrane at a point distant from the zona penetration site. Time-lapse recordings confirmed that sperm swim around the perivitelline space before fertilization. Experimental enlargement of the perivitelline space in the non-polar body half increased the regional probability of fertilization. Based on these experiments, we propose a model in which the space asymmetry exerted by the first polar body and the zona pellucida directs sperm entry preferentially to the polar body half, with no need for oocyte polarity.

## Abstract 2

The point of sperm fertilization on the mouse oocyte's surface is dictated by space constraints imposed by the encapsulating zona pellucida and first polar body; the oocyte itself is described as "asymmetrical" but "non-polarized."

## Introduction

The notion of prepatterning in the mammalian egg has long been controversial [ 1, 2]. Although the regulative capacity of the preimplantation embryos has suggested the absence of predetermination [ 3– 6], studies in the last decade have claimed its presence in the mouse egg [ 7– 10]. We recently showed that the first cleavage plane is not predetermined [ 11], and there is compelling evidence for the absence of embryonic polarity specification until the blastocyst stage [ 12– 16]. These data led to a new model in which the first embryonic axis, the embryonic-abembryonic axis, is oriented by mechanical cues exerted by the zona pellucida (ZP) in concert with the epithelial property of the outer cell layer [ 12, 15]. The question of polarity specification is not only fundamental to understanding mammalian development, but is also a crucial basis for ongoing clinical applications; preimplantation genetic diagnosis, which is based on the regulative capacity of the mammalian preimplantation embryo, and intracytoplasmic sperm injection, in which sperm is manually injected essentially randomly (except for the area close to the metaphase chromosomes), into the oocyte, both assume the absence of prepatterning.

The fully differentiated meiotic metaphase II (MII) oocyte, prior to fertilization, has often been characterized as “polarized” [ 17– 20], based on an apparent morphological asymmetry wherein the metaphase chromosomes are localized at one end (see later discussion for our definition of the term “polarity”). The cortical area overlying the meiotic spindle is specialized, enriched with actin, and lacking microvilli or sperm-binding capacity [ 21– 23]. Par3 and Par6 proteins have been recently reported to localize over the MII spindle [ 18, 20], and leptin and STAT3 have also been reported to localize asymmetrically in the oocyte [ 24], although the latter findings remain controversial [ 2]. Indeed, we found that sperm enter preferentially into the polar body (pb) half, except for the microvilli-free area directly above the metaphase chromosomes [ 11], whereas an earlier study reported that more sperm enter in the “vegetal” area [ 10]. Because this raises the possibility that preferential sperm entry is due to intrinsic polarity of the MII oocyte, we explored the mechanism of this preferential sperm entry as an avenue to determining the presence or absence of intrinsic oocyte prepatterning.

## Results/Discussion

**Figure 1:**
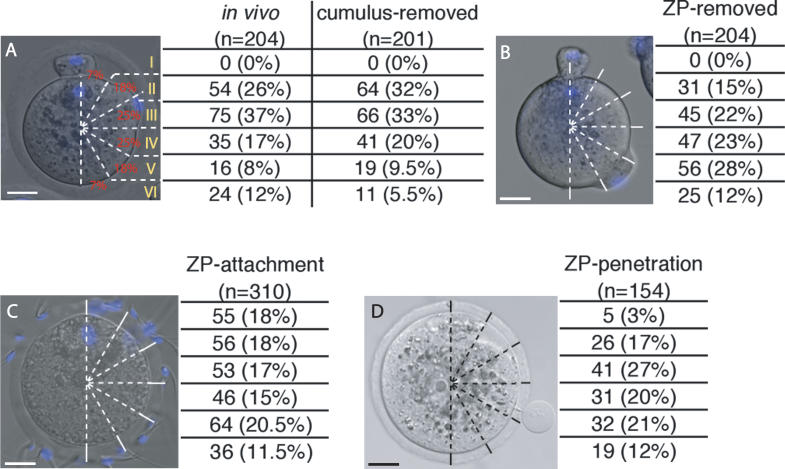
The MII Oocyte Is Preferentially Fertilized in the pb Half Sphere Independent of Intrinsic Polarity (A) Distribution of the site of fertilization with respect to the 2pb in vivo and in vitro after cumulus cell removal. Zones I–VI (depicted in yellow), defined as 30° wide each, categorize the site of fertilization with respect to the 2pb. The proportion of the surface area for each zone is depicted in red. (B) Distribution of the position of fertilization in vitro after zona pellucida removal. (C) Distribution of sperm binding to defined regions of the ZP with respect to the meiotic chromosomes at MII stage. (D) Distribution of the point of ZP penetration by the sperm with respect to the 2pb. Results are all presented in the order of Zone I to VI. Scale bars represent 20 μm.

Consistent with our preliminary data [ 11], in 63% (129 of 204) of the in vivo-fertilized eggs, sperm enters the egg in the pb half excluding Zone I (see Materials and Methods), when the fertilization cone is used as a reference point for the point of sperm entry and fertilization ( Figure 1A; and see later discussions). This is significantly higher than the rate expected from random sperm entry ( *p* < 0.0001, statistical analyses are all Chi-square analysis unless otherwise stated). The preference was preserved when oocytes with cumulus cells removed were fertilized in vitro ( *p* < 0.0001), indicating that the cumulus cell layer does not contribute to the asymmetry in the fertilization ( Figure 1A). However, when the ZP was removed by micromanipulation [ 25] ( Figure 1B; see Materials and Methods), the incidence of in vitro fertilization (IVF) in the pb half was significantly reduced (37%, 76 of 204) as compared to the in vivo-fertilized ( *p* < 0.0001) or the cumulus-free embryos ( *p* < 0.001) ( Figure 1B). The probability was in fact approximately proportional to the surface area ( *p* = 0.06; see Figure 1A and Materials and Methods; we assume based on our analysis that 11% of the area surrounding the second polar body (2pb) is incapable of sperm binding), suggesting that the sperm entry is essentially random. This uniformly distributed capacity of the oocyte membrane per se to receive sperm shows that the oocyte has no inherent polarity, at least along the pb axis, in terms of the potential for fertilization (see later discussion). Thus, we investigated further to unravel the mechanism of the preferential sperm entry in the absence of oocyte polarity.

Sperm attachment to the ZP was confirmed to be uniform ( *p* = 0.31; Figure 1C, see also Materials and Methods), allowing us to focus on the process after attachment. Using a method adapted from a previous report [ 26], we marked in vivo–fertilized egg at the ZP directly above the 2pb as a reference point, followed by a hypotonic treatment (see Materials and Methods), to reveal the point of sperm penetration through the ZP. Gradual swelling led to extrusion of cytoplasm through the tiny hole in the ZP created by sperm penetration prior to fertilization ( Video S1). The distribution of the sperm penetration points was shown to be random, proportional to the surface area ( *p* = 0.42; Figure 1D).

**Figure 2:**
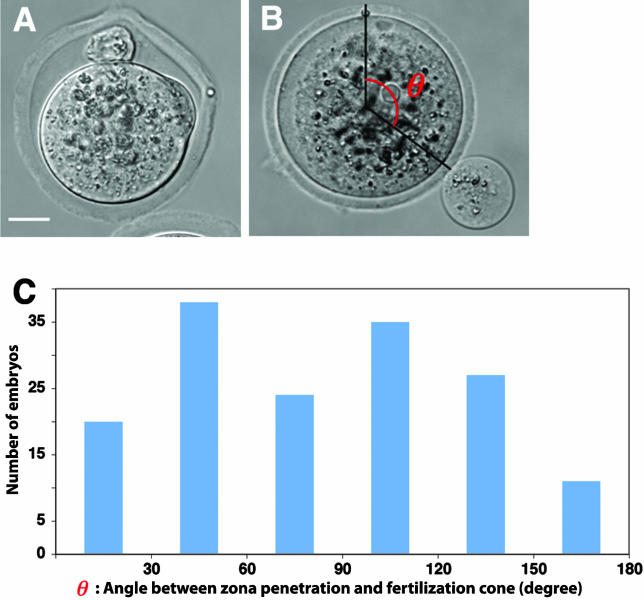
Sperm Fertilizes at a Point Far Distant from Its Site of ZP Penetration (A) Zygote with an oil drop marking the position of the fertilization cone. (B) The embryo after hypotonic treatment. The cytoplasmic protrusion marks the site of ZP penetration by the sperm. Arc distance *(θ)* between the point of ZP penetration and fertilization is measured. (C) Distribution of the arc distance between the sites of ZP penetration and fertilization. Scale bar in (A) represents 20 μm.

To investigate the apparent discrepancy between the random sperm penetration through the ZP and its preferential entry into the oocyte, we examined the topological relationship between these two events in the individual embryo. In vivo fertilized eggs were recovered when the fertilization cone was visible (15 h after human chorionic gonadotropin [hCG] injection; see Materials and Methods), and the position of the fertilization cone was marked on the ZP ( Figure 2A). Following hypotonic treatment ( Figure 2B), embryos were rotated by micromanipulator so that the point marking the fertilization cone and the point of cytoplasmic extrusion were precisely on the equatorial focal plane. The arc distance between the point of zona penetration and that of fertilization was measured for each embryo (degree *θ* in Figure 2B), and the distribution was summarized in a chart ( Figure 2C). Contrary to the previous notion [ 27], a significant proportion of the sperm fertilizes at a point far distant from that of zona penetration.

**Figure 3:**
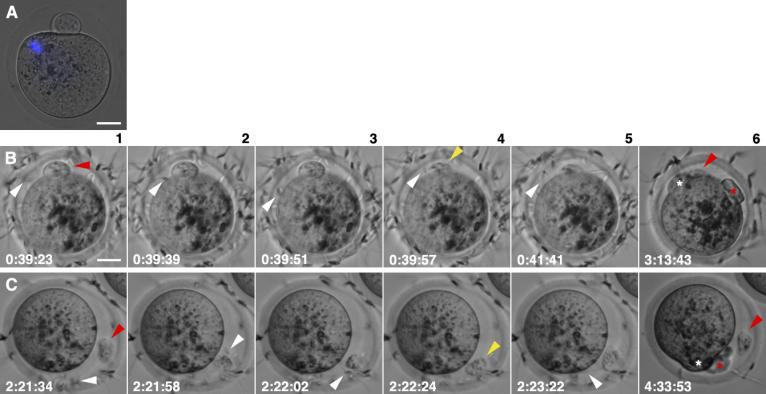
Sperm Swims Around within the PVS Prior to Fertilization (A) Typical morphology of a MII oocyte with the meiotic chromosomes located in one “shoulder.” (B and C) Sequential DIC (differential interference contrast) images of embryos during in vitro fertilization. (1) Sperm penetrates the ZP. (2 and 3) Sperm swims around the PVS. (4) Sperm hits the 1pb. (5) Sperm binds the oocyte membrane. (6) 2pb extrudes and the fertilization cone forms. White arrowheads indicate the sperm, red arrowheads the 1pb, and yellow arrowheads the collapsing (B) or rotating (C) 1pb. White and red asterisks mark the fertilization cone and the 2pb, respectively. Scale bars in (A) and (B1) represent 20 μm.

Based on our present data, we propose the following model to explain preferential sperm entry into the non-polarized oocyte. After penetrating the ZP, the sperm swims around the perivitelline space (PVS) before binding to the oocyte membrane. Most fully matured oocytes retain the intact first polar body (1pb) prior to fertilization, which is compressed between the ZP and the oocyte, and this results in a topically expanded PVS ( Figure 3A). The MII chromosomes typically reside at one of the two “shoulders” created by the compression in the oocyte (20 of 30 oocytes examined have the 1pb within 90° of the MII chromosomes, the prospective 2pb position, with an average for all oocytes of 92.1°, exemplified in Figure 3A) [ 28, 29]. This increased PVS increases the probability that the swimming sperm is present in the pb half PVS before acquiring the capacity for fertilization (see later discussion) and that it eventually binds to the oocyte membrane more frequently in the pb half. In this model, space constraints produced by the 1pb can direct the preferential sperm entry into the oocyte with no need for intrinsic oocyte polarity.

To test our model, we first filmed the IVF process to confirm that sperm swim around the PVS before binding to the oocyte membrane. We have successfully time-lapse recorded the IVF of 41 embryos starting from sperm insemination until two pronuclei formation (ranging from 5 to 7 h). All embryos were then stained for DNA to confirm the presence of two pronuclei, thus excluding polyspermy. In some embryos, the sperm was clearly observed by several independent observers to swim for 1 to 3 min within the PVS ( Figure 3B and 3C, and the corresponding Videos S2 and S3), although the extensive activity of the sperm prevented tracing each and every movement using time-lapse recording (every 2 s). In some cases, the swimming sperm hit the 1pb and induced its abrupt degeneration ( Figure 3B4, Video S2) or quickly rotated it backward and forward ( Figure 3C2–4, Video S3). Most of the oocytes showed some jiggling movement between the moment of the zona penetration and that of fertilization ( Video S3). The moment of fertilization was relatively easily identified by a short contraction, most likely associated with the Ca ^2+^ wave, which was usually preceded by 30–60 s by the sperm attachment to the oocyte membrane ( Video S3).

These movies also revealed a dynamic cytoplasmic rearrangement associated with fertilization, including the movement of surface membrane area such as the point of sperm attachment and that of the 2pb extrusion. Although this movement might influence the interpretation of the conventional method employed in the current study to identify the point of fertilization based on that of the fertilization cone, our movies showed that these two points are usually very close to each other and the latter can be reliably used as a reference for the former: It was indeed the case for four out of five movies in which both points are located in close proximity to the focal plane and are reliably identified by several independent observers. Thus, this dynamic cytoplasmic rearrangement may possibly contribute only partially, if at all, to the preferential formation of the fertilization cone in the pb half.

Previous studies (reviewed in [ 27]) proposed a model in which the oocyte spins inside the ZP, pushed along by the swimming sperm following its attachment. Several time-lapse studies [ 30– 32] showed that in a few embryos, fertilization took place several minutes after zona penetration and the sperm wandered in the PVS and/or rotated the oocyte, but such embryos were considered exceptional and their significance was dismissed. Unfortunately, these claims were supported by a very limited number of observations [ 33], and the location of the zona penetration or of the fertilization (i.e., sperm attachment) was not specified, making it difficult to directly compare them with our present results. Although our observation of incongruity between the point of zona penetration and that of fertilization might be explained by the putative spinning of the oocyte, we consider this possibility very unlikely, since only one of 41 embryos (2%) examined in the present time-lapse analysis showed the oocyte spinning more than 90° after sperm attachment. In fact, in seven of 23 embryos (30%) examined under our time-lapse recording conditions, the fertilization point was more than 90° distant from the point of ZP penetration. The lower frequency than in in vivo conditions (47%, 73 of 155; Figure 2C) is likely due to reduced sperm motility under the suboptimal recording condition. Thus, the previous spinning model [ 27] cannot explain the majority of the cases in which the sperm fertilizes far from the ZP penetration point.

**Figure 4:**
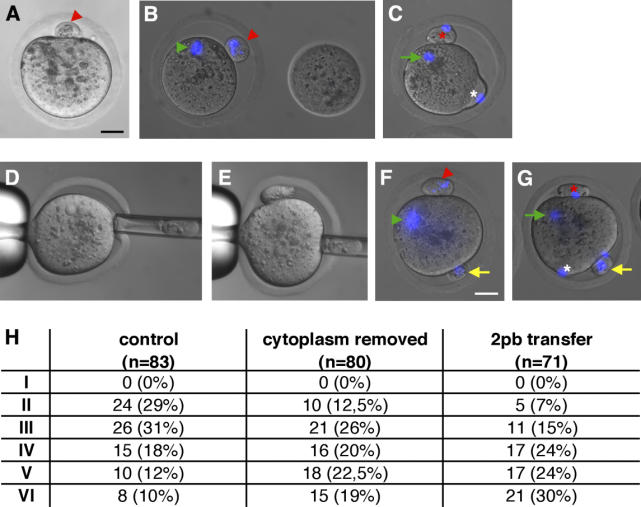
Experimental Enlargement of the PVS in the Non-pb Half Increases the Probability of Fertilization in This Area (A–C) Removal of cytoplasm at MII stage and fertilization in non-pb half of a manipulated embryo. (A) MII oocyte. (B) Manipulated oocyte (left) following removal of half of its original volume (right). (C) Fertilized zygote. (D–G) 2pb transplantation and fertilization in non-pb half of manipulated embryo. (D) 2pb removal from donor zygote. (E) 2pb transfer to another oocyte. (F) MII oocyte with additional 2pb and PVS. (G) Fertilized zygote. (H) Classification of embryos according to their site of fertilization with respect to the 2pb under control and manipulated conditions. Zones I–VI are defined as in Figure 1. Red arrowheads indicate the 1pb, red asterisks indicate the 2pb, white asterisks indicate the fertilization cone, green arrowheads indicate MII chromosomes, green arrows indicate condensed female chromosomes, and yellow arrows indicate the transplanted 2pb. Scale bars in (A) and (F) represent 20 μm.

Our space constraints model predicts that additional PVS, locally imposed by experimental manipulation, would increase the regional probability of fertilization ( Figure 4). Experiments in which we removed approximately half of the cytoplasm ( Figure 4B) or transplanted a 2pb from one zygote ( Figure 4D) into the PVS of another MII oocyte ( Figure 4E) produced additional PVS in the non-pb half in both cases ( Figure 4B and 4F). The embryos were fertilized in vitro, collected when they formed the fertilization cone, and stained for DNA to confirm proper fertilization ( Figure 4C and 4G). The results summarized in Figure 4H indicate a significant increase ( *p* = 0.006 and *p* < 0.0001) in the probability of fertilization in the non–pb half (Zones IV, V, and VI) under both experimental conditions, whereas no significant increase was found in the control embryo in which only the slit is made in the ZP, thus strongly supporting our model.

Taken together, these data show that preferential sperm entry during fertilization does not require any intrinsic polarity in the oocyte. We propose a model in which the space constraints in the oocyte exerted by the ZP and 1pb lead to an increased probability that the swimming sperm localizes and eventually enters into the pb half where more PVS is available. Fertilization is considered to be a two-step process of sperm adhesion and fusion [ 33, 34]. Although CD9 was shown to be required for sperm fusion and to be expressed with no apparent asymmetry on the oocyte surface [ 35, 36], the molecules essential for the sperm adhesion remain largely unknown. Our model predicts that such molecules, when found, will be expressed uniformly on the oocyte surface (except for the area directly above the metaphase chromosomes). This model also raises the possibility that sperm require additional modifications to obtain the capacity to fertilize the oocyte membrane following the zona penetration, which could well explain why the sperm swims around in the PVS for the time required to achieve this modification. Another possible mechanism might be that simple contact between the oocyte surface and the sperm head is not sufficient and some free space is required, or preferred, to trigger the fusion of the gametes.

Here, we propose to define the term embryonic “polarity” as a morphological and/or molecular “asymmetry” *with relevance to the future embryonic patterning,* in the sense that *Drosophila* and *Xenopus* eggs are polarized. Because no polarity specification in that sense, i.e., prepatterning of the blastocyst embryonic-abembryonic axis, could be found from the (fertilized) egg until the blastocyst stage [ 11– 16], and because the cortical migration of the meiotic chromosomes is unpredictable [ 37], it is reasonable to consider the fully differentiated mouse MII oocyte as “asymmetrical,” due to the polar body exclusion necessary for meiosis, but “non-polarized.” The cortical microvilli-free area could represent a specialized area inherent to the process of meiotic division, to be excluded as a 2pb. This may also explain the localization of Par-3 and Par-6 proteins in this specialized area over the MII spindle [ 18, 20]. The idea of complete absence of polarity at all stages ranging from immature oocyte to the blastocyst stage is consistent with ongoing practices in reproductive medicine, e.g., preimplantation genetic diagnosis and intracytoplasmic sperm injection, and provides a rational conceptual basis for understanding mammalian development.

## Materials and Methods

### Embryo collection and in vitro fertilization

For all experiments, MII oocytes were recovered from superovulated (C57BL/6xC3H) F1 female mice, and zygotes were from superovulated female mice mated with (C57BL/6xC3H or C57BL/6xDBA/2) F1 male mice 13.5–14.0 h after hCG(5 IU) injection. After brief treatment with hyaluronidase (300 U/ml), oocytes or zygotes were washed with H-KSOM-AA (KSOM with amino acids and 21 mM HEPES) several times and cultured prior to experiments in a microdrop of H-KSOM-AA covered with mineral oil (Acros Organics, Fair Lawn, New Jersey, United States) in 6.3% CO _2_ at 37.5 °C. To obtain ZP-free oocytes ( Figure 1B), the ZP was slit with a needle [ 25] and the oocyte was aspirated using a 40-μm diameter pipette in H-KSOM-AA. Although ZP-free oocytes tend to stick to the bottom of the culture dish employed for the IVF, thus possibly influencing the regional probability of fertilization, this should most likely not introduce any bias because the attachment to the bottom occurs at a random point of the entire oocyte surface. Sperm was isolated from the cauda epididymis of (C57BL/6xDBA/2 or C57BL/6xC3H) F1 male mice and capacitated in a 300-μl drop of human tubal fluid (HTF) with 4 mg/ml BSA covered with mineral oil in 6.3% CO _2_ at 37.5 °C for 1.5–2 h. The concentration of sperm was determined using a Neubauer hemacytometer. Depending on the experimental condition, various concentrations of sperm were used for insemination, i.e., after cumulus removal, 5 × 10 ^5^ sperm/ml; after ZP removal, 5 × 10 ^4^ sperm/ml; and for time-lapse recordings, 2.5 × 10 ^5^–1×10 ^6^ sperm/ml. IVF was performed in a 250-μl drop of HTF-containing BSA covered with mineral oil. For time-lapse recordings, no BSA was added to HTF to allow adherence of the oocytes to the bottom of the dish. To identify the fertilization cone position, embryos were collected 15–16 h post-hCG, or 2–3 h after IVF, depending on the experimental condition.

### Time-lapse recording of the embryos

Time-lapse recordings were started immediately after insemination of 10 to 15 oocytes. Temperature was maintained by a Tempcontrol 37–2 digital (Carl Zeiss, Oberkochen, Germany) at 37.5 °C in a plastic chamber incubator XL (Zeiss) and a heatable mounting frame M-H (Zeiss), attached to a Zeiss Axiovert 200M with Narishige manipulators. The CO _2_ concentration was regulated by a CO _2_ controller (Zeiss) connected to a humidifier and a CO _2_-cover KH (Zeiss). To observe the expanded PVS surrounding the 1pb and formation of the 2pb, the manipulator was used to orient the oocyte at the beginning so that the 1pb and the meiotic chromosomes were in the same focal plane. Zeiss AxioVision Ver. 4.3 software was used for the acquisition of the time-lapse images. The halogen lamp was set below 2.4 V to minimize the embryo's exposure to light. Embryos were recorded every 2–10 s for up to 7 h. Under this condition, initiation of zona penetration was observed about 80 min after insemination, followed by a cytoplasmic contraction indicative of fertilization in 10 additional minutes. Extrusion of the 2pb was initiated soon after the fertilization and was readily detectable due to increased movement of the cytoplasm. About 2.5 h after insemination (1 h after fertilization), the fertilization cone appeared at the site of sperm fusion. To confirm monospermy, number of sperm entering in the PVS, and appropriate pronuclear formation, embryos were stained for DNA with bisBenzimide (Hoechst 33258) (10 μg/ml; Sigma, St. Louis, Missouri, United States) or DAPI (4',6-Diamidino-2-phenyindole) dilactate (10 μM; Sigma) immediately after every recording. The average efficiency of fertilization was 34% under this recording condition. The time-lapse movies were converted to QuickTime movies using ImageReady (Adobe, San Jose, California, United States).

### Analysis of the topological relationship of pbs, points of zona penetration, and fertilization

To determine the position of sperm penetration through the ZP, zygotes were incubated in 0.5% sodium citrate dihydrate (Mallinckrodt Baker, Phillipsburg, New Jersey, United States) for a few minutes until part of the cytoplasm protruded through a tiny hole in the ZP that was introduced by the penetrating sperm ( Figures 1D and 2B). To map the position of the 2pb or the fertilization cone prior to hypotonic treatment, oil drops were injected into the ZP directly overlying these structures [ 8, 15] ( Figures 1D, 2A, and 2B). One drop marks the 2pb and two drops, the fertilization cone. For fixation, embryos were incubated in 4% paraformaldehyde (PFA; Sigma) in PBS for 10 min at room temperature. To exclude polyspermic embryos, embryos were stained for DNA as described above. For documentation and analysis, embryos were rotated using a micromanipulator to align the points of interest, i.e., the meiotic spindle, 2pb, fertilization cone, and the site of sperm penetration, in the focal plane traversing the equatorial plane. By contrast, analysis of the sperm zona attachment ( Figure 1C) was carried out for the sperm bound close to the focal plane. To determine the spatial relationship of these points, manipulated eggs were always subdivided into six zones (Zones I–VI) representing horizontal discs of 30° each ( Figure 1A). Zone I is the closest to the 2pb, whereas Zone VI the furthest from the 2pb. Meiotic spindle, 2pb, fertilization cone, and sperm penetration site were assigned to a specific Zone depending on the central point of these structures. The minimal arc distance between the MII chromosomes/2pb and the fertilization cone in the samples of Figure 1A was 38°, providing us with the estimate that the sperm cannot fertilize in the 11% surface area surrounding the polar body. The surface area is calculated at the macroscopic level, and contribution of the microvilli is not taken into account. To determine the spatial relationship of the fertilization cone and the position of sperm penetration through the ZP, six subdivisions of 30° each were made to span 180° with the position of fertilization cone as a reference point ( Figure 2C).

### Experimental manipulations

Two strategies were applied, removal of cytoplasm and transplantation of an additional 2pb, to increase PVS. For cytoplasmic removal, a slit was made in the ZP of MII oocytes on the opposite side of the MII spindle. Half of the cytoplasm was removed using a pipette of 20-μm diameter in a microdrop of H-KSOM containing Cytochalasin B (5 μg/ml; Sigma) ( Figure 4B). The 2pb from another zygote was introduced through a slit in the ZP into the opposite side of the MII spindle of the recipient oocyte ( Figure 4D–F). Control embryos were prepared in which the slit was made in the ZP in the same way as for the manipulated embryos, but no manipulations were carried out.

## Supporting Information

Video S1 This Video Shows Zygotes Swelling after Exposure to 0.5% Sodium Citrate Dehydrate Swelling results in the partial extrusion of the plasma membrane and cytoplasm through the ZP at the site of sperm penetration.
(1.6 MB MOV)

Video S2 Sperm Movement in the PVS during In Vitro Fertilization, as Described in Figure 3B
White arrowhead depicts the sperm. White exclamation mark depicts the time of fertilization, identified by a short cytoplasmic contraction. Black asterisk marks the site of 2pb formation, white asterisk the site of fertilization cone formation. Time given in hh:mm:ss represents real time. This video consists of 1,392 frames; 20 msec (frames 1–744), 80 msec (frames 745–1,196), or 100 msec per frame (frames 1,197–1,392), with constant relative speed.
(4.3 MB MOV)

Video S3 Sperm Movement in the PVS during In Vitro Fertilization, as Described in Figure 3C
White arrowhead depicts the sperm. White exclamation mark depicts the time of fertilization, identified by a short cytoplasmic contraction. Black asterisk marks the site of 2pb formation, white asterisk the site of fertilization cone formation. Time given in hh:mm:ss represents real time. This video consists of 871 frames; 20 msec (frames 1–464), 80 msec (frames 465–690), or 100 msec per frame (frames 691–871), with constant relative speed.
(5.1 MB MOV)

## Abbreviations

1pb: first polar body
2pb: second polar body
hCG: human chorionic gonadotropin
HTF: human tubal fluid
IVF: in vitro fertilization
MII: meiotic metaphase II
pb: polar body
PVS: perivitelline space
ZP: zona pellucida
